# Referral rate for refractive amblyopia using automated vision screening in school children in Beirut, Lebanon

**DOI:** 10.1371/journal.pone.0323361

**Published:** 2025-05-12

**Authors:** Zahi Wehbi, Hanadi Ibrahim, Youssef Zougheib, Zeinab El Moussawi, Dalia El Hadi, Christiane Al-Haddad

**Affiliations:** 1 Ophthalmology Department, American University of Beirut, Beirut, Lebanon,; 2 American University of Beirut Medical Center, Beirut, Lebanon; Alexandria University Faculty of Medicine, EGYPT

## Abstract

**Background:**

Amblyopia is one of the most prevalent causes of decreased vision in children and can be effectively diagnosed at an early stage through vision screening. Untreated vision impairments during childhood have lasting implications on academic achievements. The purpose of this study was to compare referral rates when applying different referral criteria including the AAPOS 2021 exam failure levels and the Arnold “medium” 2022 Instrument Referral Criteria.

**Methods:**

Automated vision screening was conducted in four selected schools (two private and two public) in Beirut, Lebanon. Children aged 3–6 years old were targeted. The Plusoptix A12 Refractometer was utilized to perform vision screening. Referral rate was computed for the American Association of Pediatric Ophthalmology and Strabismus (AAPOS) age-based criteria applied to the Plusoptix A12 and compared to the Arthur exam failure criteria and 4 instrument referral criteria (Arnold Medium and Specific, Matta & Silbert, and Alaska Blind Child Discovery). Referral rates were also compared between public and private schools based on the AAPOS criteria.

**Results:**

A total of 308 children were screened: 114 students from public schools and 194 students from private schools. The gender distribution in the two groups was similar (46% females in public schools and 48% in private schools); 34% of the studied population were under 4 years old, while 66% were ≥4 years. The referral rate using the AAPOS 2021 criteria was 22%. There was a significant difference in referral rates overall and across the different types of refractive error when compared with the other referral criteria. Referral rate using the ABCD criteria was similar (17.9%). Referral rates were higher when applying Arthur criteria (28.2%) and Matta & Silbert criteria (41.9%), while the Arnold Medium and Specific criteria had the lowest referral rates (11.4%, 9.1%). The most common refractive error across all criteria was astigmatism. The overall referral rate using the AAPOS 2021 criteria was 22% and this differed significantly by school type, with a rate of 36% for public schools and 14% for private schools (p < 0.001). Spherical equivalent was also higher in public schools. Among children needing referral, 40.6% were already wearing spectacles at the time of examination and this differed by school type (4.9% of children referred from public schools and 92.8% of children referred from private schools).

**Conclusion:**

Photoscreener referral rate and the detection of the different types of refractive errors varies significantly according to the referral criteria used. Careful consideration of psychometric characteristics of referral criteria is important and the use of device-specific age-based criteria is recommended. In our cohort, the most common refractive error was astigmatism. The overall rate of referral was 22% according to the AAPOS age-based exam failure referral criteria, which differed from referral rates applying other validated instrument referral criteria in the literature. The referral rate and spherical equivalent were higher in public schools compared to private schools.

## Background

Early childhood vision screening is crucial to detect preventable and treatable vision disorders [1]. Amblyopia is the most common cause of reduced vision in children, but early diagnosis through vision screening and referral for complementary exams, and early intervention can improve visual outcomes. National screening programs have reduced the burden of amblyopia in some countries [2]. Undiagnosed and untreated vision impairments in childhood can hinder learning and lead to long-term consequences for school success [3]. The US Preventive Services Task Force recommends that all children get automated screening to detect amblyogenic risk factors and refractive errors timely [1]. The American Academy of Pediatrics recommends screening for distance visual acuity, ocular alignment, and ocular media clarity for children 3–6 years of age and older [4,5]. The American Academy of Ophthalmology and the American Association for Pediatric Ophthalmology and Strabismus recommend vision screening during the preschool years [3]. Studies have confirmed the effectiveness of the Plusoptix A12 refractometer in detecting visual impairment [6]. The screener was found to be effective in detecting amblyopia, strabismus, and refractive errors in children as young as 3 years old [6]. Cycloplegic retinoscopy and clinical examination confirm the diagnosis in suspected cases [7].

A recent study using school-based photoscreening analyzed data from over 123,000 children and found a significant increase in myopia in younger children aged 6–8 years in 2020 compared to previous years (2015–2019) [8]. Showing good consistency with the cycloplegic refraction test, the Spot Vision Screener used was considered a reliable myopia screening tool [8]. Other studies during the COVID-19-related lockdown showed that children aged 3–10 years had more myopia when compared to the pre-lockdown period [9]. Myopia rate in China increased by 10% between 2019 and 2020, especially in children exposed to smartphones and computers [10]. Saara et al. found that the prevalence of vision impairment, refractive errors, and myopia in at least one eye was 12.83%, 21.51%, and 19.53%, respectively [11].

Importantly, photoscreening referral rates and device performance are significantly affected by the screening criteria used [12]. The American Association of Pediatric Ophthalmology and Strabismus (AAPOS) specifies age-based criteria for the confirmatory cycloplegic examination levels to inform the development and validation of screening devices and referral criteria [13]. In a study by Yan et al., the AAPOS criteria applied to the Plusoptix was found to have a sensitivity of 46.34% and a specificity of 98.93% [14]. Multiple other sets of referral criteria are proposed in the literature based on studies targeting different thresholds of device performance. Five of the most used sets of criteria in the literature are those proposed by Arnold (Medium and Specific, 2022), Matta & Silbert, Arthur, and the Alaska Blind Child Discovery (ABCD) project. Arnold et al. in 2021 evaluated the performance of three photoscreeners, including the Plusoptix, and proposed referral criteria for maximum accuracy, in addition to criteria with higher specificity or sensitivity while maintaining accuracy [12]. Matta & Silbert in 2008 proposed revised referral criteria for the Plusoptix achieving a sensitivity of 98% and specificity of 88% [15]. Arthur et al. in 2008 found a sensitivity of 83% and specificity of 95% for their criteria [16]. Table 1 summarizes the referral criteria applied in this study: the AAPOS exam failure criteria, the Arthur exam failure and 4 instrument referral criteria (IRC) proposed in the literature (Arnold Medium and Specific, Matta & Silbert, and Alaska Blind Child Discovery).

**Table 1:** The sets of referral criteria applied in the study.

	Hyperopia	Myopia	Astigmatism	Anisometropia
**Exam failure criteria used as Instrument Referral Criteria**				
*AAPOS 2021*				
<4 years	≥4.00 D	≤-3.00 D	≥3.00 D	≥1.25 D
≥4 years	≥4.00 D	≤-2.00 D	≥1.75 D	≥1.25 D
*Arthur*	≥3.50 D	≤-3.00 D	≥1.25 D	≥1.0 D
**Instrument Referral Criteria**				
*Arnold Medium* ^*^				
<4 years	≥3.00 D	≤-3.50 D	≥3.50 D	≥1.75 D
≥4 years	≥3.00 D	≤-2.50 D	≥2.50 D	≥1.75 D
*Arnold Specific* ^**^				
<4 years	≥3.50 D	≤-3.50 D	≥3.75 D	≥1.75 D
≥4 years	≥3.50 D	≤-2.50 D	≥2.75 D	≥1.75 D
*Matta & Silbert*	≥1.25 D	≤-1.00 D	≥1.00 D	≥1.25 D
*Alaska Blind Child Discovery*	≥2.50 D	≤-2.25 D	≥2.25 D	≥1.00 D

*D: diopters.*

*: The ideal medium instrument referral criteria for maximal accuracy reported by Arnold [12].

**: The specific instrument referral criteria reported by Arnold [12].

Overall, studies demonstrate that refractive errors are a significant public health issue among school-aged children, with increasing prevalence rates reported in various countries [6–11]. Early detection and intervention are essential to prevent lifelong visual impairment and related complications [1,5]. Despite the importance of early detection and treatment of refractive errors, there is lack of research on the prevalence of these conditions in the Middle East, particularly in Lebanon. A previous study in a hospital-based cohort in our institution showed a 5.7% rate of refractive amblyopia risk factors with hyperopia being the most common [17]. Our current study aims to detect the rate and type of refractive amblyopia risk factors in a community-based cohort of Lebanese children aged 3–6 years attending schools in the Beirut area by applying the AAPOS age-based exam failure criteria to the Plusoptix A12 and comparing them to the Arthur exam failure criteria and 4 IRC proposed in the literature with sub-comparison by school type.

## Materials and methods

This was a school-based vision screening conducted in four schools in the Beirut area (2 private and 2 public). The study was approved by the American University of Beirut institutional review board (IRB) (ID: BIO-2019–0109). Additionally, the study team obtained approval from the Ministry of Education and the schools’ principals. All methods were carried out in accordance with the declaration of Helsinki. Written informed consent was obtained and signed by the parents and/or legal guardians for participation in the study and appointments were scheduled for vision screening. The study targeted children aged 3–6 years, including nursery, kindergarten 1, 2, and 3, and grade 1. Screening using the Plusoptix A12 Refractometer was performed for children whose parents provided signed consent. Data was collected from November 1, 2022, until May 31, 2023.

Vision screening was performed by trained MD personnel from the ophthalmology department (authors ZW and HI). Data on refractive error were extracted from the photo-screener and later analyzed. In addition, information on grade, gender, and whether the student wore spectacles were collected and stored in an excel database.

### Vision screening (1–5 minutes)

PlusoptiX A12 Refractometer (Plusoptix GmbH, Atlanta, GA), software version 7.2.6.0, was used as a sensitive vision screening tool. Performing vision screening was a simple and fast process that took around 1–5 minutes only to detect refractive error. The researcher/examiner stood at least 1 meter away from the student, and no physical contact was needed. The camera was set off by pushing a button. The sound attracted attention, and both eyes were captured on screen in a white rectangle and a measurement was automatically registered, with results immediately displayed on the screen. The device could also detect any strabismus or media opacities. The test was repeated at least twice when results showed need for referral or “MYO/ HYP” on the screen, signifying high myopia and hyperopia respectively.

### Referral

This study aimed to determine the rate of refractive amblyogenic risk factors among school-aged children, including hyperopia, myopia, astigmatism, and anisometropia, based on AAPOS 2021 age based examination failure criteria [13] applied as IRC to the Plusoptix A12, the Arthur exam failure criteria [16], and 4 validated IRC from the literature proposed by Arnold (Medium and Specific) [12], Matta & Silbert [15], and the ABCD project [18]. We also sought to compare results between public and private schools. The different referral criteria are represented in Table 1. If a significant refractive error was encountered based on the AAPOS 2021 criteria, a referral letter was sent to parents.

### Statistical analysis

The data were analyzed using IBM SPSS (Statistical Packages for Social Sciences) version 28. A sample size of at least 216 participants was calculated using an estimated proportion of 0.17 for the prevalence of refractive errors in school children from previous studies in the Middle East and using the formula for determination of a single sample proportion with a margin of error of 0.05 and a 95% confidence interval [19]. Categorical variables were reported in frequencies and proportions, and the Chi-square test was used to assess differences between groups. If more than 25% of expected cells had less than 5 observations, Fischer’s exact test was performed. The Shapiro-Wilk test of normality was used to evaluate the normality of continuous variables. For normally distributed data, the mean and standard deviation were reported, and a t-test was used to compare two groups. Cochran’s Q test was used to compare referral and refractive error rates across different criteria. The McNemar test was used for pairwise post-hoc comparisons. The statistical significance level was set at a p-value of less than 0.05.

## Results

### Demographics

A total of 308 students participated in the study that included 114 children from public schools and 194 from private schools, (616 eyes). The participants were divided into two groups based on the type of school they attended, public or private. Table 2 provides information on the age, grade, and gender of the participants.

**Table 2:** Demographics of all study participants (n = 308 students; 616 eyes).

	All	Public	Private	p-value
**Number (n)**	308	114	194	
**Age (years)**				
<4	106 (34%)	13 (11%)	93 (48%)	<0.001
≥ 4	202 (66%)	101 (89%)	101 (52%)	
**Grades**				
Nursery	46 (15%)	–	46 (24%)	
Kindergarten 1	60 (19%)	13 (11%)	47 (24%)	
Kindergarten 2	96 (31%)	22 (19%)	74 (38%)	
Kindergarten 3	67 (22%)	40 (35%)	27 (14%)	
Grade 1	39 (13%)	39 (34%)	–	
**Gender**				
Female	146 (47%)	52 (46%)	94 (48%)	0.64
Male	162 (53%)	62 (54%)	100 (52%)	

The distribution of participants by grade is presented in Table 2. Among the public-school students, the majority (n = 101, 89%) were 4 years or older. In contrast, among the private school students, 52% were 4 years or older. The difference between the two groups was statistically significant (p < 0.001). Gender distribution was similar: females constituted 46% in public schools and 48% in private schools (p = 0.64).

### Referral rates by criteria

Table 3 shows the referral rates and rates of hyperopia, myopia, astigmatism, and anisometropia when AAPOS and each set of criteria were applied to the results of the Plusoptix A12. In terms of overall referral rate, there was a significant difference between all criteria (p < 0.001). The referral rate using the AAPOS criteria was 22%. Pair-wise post-hoc testing showed that the ABCD criteria had a similar rate of 17.9% (p = 0.61). The Arnold Medium and Arnold Specific criteria had significantly lower referral rates (11.4% and 9.1%, p < 0.001). The Arthur criteria (28.2%) and the Matta & Silbert criteria (41.9%) both had a significantly higher referral rate than the AAPOS criteria (p < 0.001).

**Table 3:** Referral rate and rates of different refractive errors according to the different referral criteria.

Criteria	Referral Rate n (%)	Hyperopia n (%)	Myopia n (%)	Astigmatism n (%)	Anisometropia n (%)
**Exam failure criteria used as Instrument Referral Criteria**					
AAPOS 2021	69 (22%)	7 (2%)	14 (5%)	37 (12%)	14 (4.7%)
Arthur	87 (28.2%)	10 (3.2%)	5 (1.6%)	69 (22.4%)	17 (5.7%)
**Instrument Referral Criteria**					
Arnold Medium	35 (11.4%)	11 (3.6%)	4 (1.3%)	20 (6.5%)	5 (1.7%)
Arnold Specific	28 (9.1%)	10 (3.2%)	4 (1.3%)	14 (4.5%)	5 (1.6%)
Matta & Silbert	129 (41.9%)	38 (12.3%)	26 (8.4%)	93 (30.2%)	14 (4.7%)
Alaska Blind Child Discovery	55 (17.9%)	11 (3.6%)	10 (3.2%)	30 (9.7%)	17 (5.5%)
Cochran’s Q p-value^*^	<0.001	<0.001	<0.001	<0.001	<0.001

*: p-value for Cochran’s Q test comparing overall referral rate and rates of refractive error across all criteria.

In terms of type of refractive error, there was also a significant difference among the studied sets of criteria across all types of errors (p < 0.001). For hyperopia, the AAPOS 2021 criteria had a rate of 2%. The Arthur criteria (3.2%, p = 0.25), Arnold Medium (3.6%, p = 0.13), Arnold Specific (3.2%, p = 0.25), and ABCD (3.6%, p = 0.13) had similar rates. The Matta & Silbert criteria lead to a significantly higher rate of hyperopia (12.3%, p < 0.001). For myopia, the AAPOS criteria had a rate of 5%. The ABCD criteria had a similar rate (3.2%, p = 0.38). Arnold Medium and Specific (1.3%, p = 0.004) and Arthur (1.6%, p = 0.008) had lower rates, while Matta & Silbert (8.4%) lead to a significantly higher rate of myopia (p < 0.001). The rate of astigmatism based on the AAPOS criteria was 12%, which was higher than that applying Arnold’s Medium (6.5%, p < 0.001), Arnold Specific (4.5%, p < 0.001), and ABCD (9.7%, p = 0.01) criteria but lower than those by Arthur (22.4%) and Matta & Silbert criteria (30.2%) (p < 0.001). For anisometropia, AAPOS criteria detected a rate of 4.7%, similar to those by Matta & Silbert (4.7%, p = 1), ABCD (5.5%, p = 0.25), and Arthur (5.7%, p = 0.06). Arnold’s Medium and Specific criteria detected a significantly lower rate of anisometropia (1.7% and 1.6%, p = 0.004).

### Refractive errors in public and private schools

Based on the AAPOS 2021 criteria, 22% of all children needed referral and 9% were already wearing glasses. The prevalence of astigmatism was 12%, followed by myopia (5%) and hyperopia (2%).

When divided by school type, the rate of referral was 36% in public schools and 14% in private schools (p < 0.001), as shown in Table 4. The difference in rates of all detected refractive errors between public and private schools was statistically significant, being always higher in public school children (4% vs 1% hyperopia, 7% vs 3% myopia and 22% vs 6% astigmatism). The mean astigmatism power in diopters tended to be higher among public school children (2.18 ± 0.69 D) compared to private school children (1.94 ± 0.87 D). The difference in spherical equivalent (SE) was statistically significant, with a mean of + 0.45 ± 0.67 D among private school children and + 0.82 ± 1.00 D among public school children (p < 0.001). With respect to anisometropia, this was detected in 2% of the whole group and this was similar comparing private and public schools. Regarding spectacle use, only 2% of public-school children wore glasses compared to 14% of private school children (p < 0.001). Among children needing referral, 40.6% were already wearing spectacles: 92.8% in private and only 4.9% in public schools (p < 0.001). All 28 children who were already wearing spectacles at the time of screening were referred for full examination to determine if a change in prescription was needed. Table 4 presents data on refractive errors, spectacle wear, and referrals among public and private school children, based on AAPOS criteria. Among our participants, 10 students (3.2%) had no reading on the PlusoptiX; all these students were referred for further ophthalmic examination; 2 students showed a low level of cooperation and refused to undergo screening. The photoscreener displayed “HYP” signifying hyperopia >5.00 diopters for 7 students, and “MYO” (myopia) for 1 student.

**Table 4:** Refractive error, spectacle wear, and referrals among public and private school children based on the AAPOS 2021 referral criteria.

	All	Public	Private	p-value
**Number (n)**	308	114	194	
**Referral n (%)**	69 (22%)	41 (36%)	28 (14%)	<0.001
**Glasses**	28 (9%)	2 (2%)	26 (14%)	<0.001
**Refractive error n (%)**				
Hyperopia	7 (2%)	5 (4%)	2 (1%)	0.010
Myopia	14 (5%)	8 (7%)	6 (3%)	0.028
Anisometropia	6 (2%)	1 (1%)	5 (3%)	0.226
Astigmatism	37 (12%)	25 (22%)	12 (6%)	<0.001
**Mean ±** **SD (diopters)**				
Hyperopia^*^	–	–	–	–
Myopia	-2.21 ± 1.41	-2.09 ± 1.53	-2.35 ± 1.27	0.64
Astigmatism	2.10 ± 0.76	2.18 ± 0.69	1.94 ± 0.87	0.23
Spherical equivalent	0.59 ± 0.83	0.82 ± 1.00	0.45 ± 0.67	<0.001

*For hyperopia requiring referral according to AAPOS criteria (Sphere >4 D), the screener displayed “HYP” signifying hyperopia >5.00 D

## Discussion

This study investigated the rate of refractive amblyopia risk factors (ARF) using automated vision screening in children aged 3–6 years attending schools in the Beirut area. We reported referral rate based on the AAPOS 2021 age-based cycloplegic examination failure rate applied to the Plusoptix A12 refractive values and compared the overall referral rate and rates of each type of refractive error to the Arthur exam failure criteria and 4 validated IRC in the literature, including Arnold Medium and Specific, Matta & Silbert, and ABCD. We also compared outcomes between public and private schools. Referral rates varied significantly between criteria. The referral rate using the AAPOS 2021 criteria was 22% when applied to Plusoptix absolute refractive values. The referral rate using the ABCD criteria was similar (17.9%). The referral rates using the Matta & Silbert criteria (41.9%) and Arthur (28.2%) were significantly higher than the AAPOS criteria. Arnold’s Medium and Specific criteria (11.4% and 9.1%) had the lowest referral rate. The most common refractive error for all criteria was astigmatism, at 12% using the AAPOS criteria which differed significantly from Matta & Silbert (30.4%), Arthur (22.4%), ABCD (9.7%), Arnold Medium, (6.5%), and Arnold Specific (4.5%). With similar gender distribution, a significantly higher referral rate using the AAPOS 2021 criteria (36%) was noted in public when compared to private schools (14%). In addition, public school children had higher SE refraction than those attending private schools. Importantly, among children needing referral, only 40.6% were already wearing glasses with a significant difference between school types: 92.8% in private schools vs only 4.9% in public schools. The prevalence of refractive amblyogenic risk factors (ARFs) reported in the literature varies by the cut-off limits followed, method used (automated screening vs acuity testing), region where the study was performed, ethnicity, and age group studied. Several studies have highlighted the importance of automated vision screening in detecting refractive errors and amblyogenic risk factors [4,5].

A review of 18 papers showed a sensitivity range between 47% and 99% for the Plusoptix, with a specificity ranging from 49 to 100% [20]. The Plusoptix A12 detected efficiently ocular abnormalities such as strabismus, amblyopia, and refractive errors in children as young as 3 years old [4]. Moreover, the Plusoptix A12C showed accuracy in detecting refractive ARFs in preschool Chinese children aged 3–4 years [5]. Multiple other papers in the literature compared different referral criteria applied to the same photoscreener device, including the Plusoptix. Yan et al. sought to evaluate the AAPOS 2021 and Arnold criteria applied to the Plusoptix against gold standard cycloplegic refraction [14]. Both criteria were highly specific (AAPOS 2021: 98.93%, Arnold: 99.38%), but had low sensitivity (AAPOS 2021: 46.34%, Arnold: 24.39%). In our study, the Arnold Specific criteria had the lowest overall referral rate (9.1%) followed by the Arnold Medium criteria (11.4%), since the thresholds were higher than exam failure rates set by AAPOS for myopia, astigmatism and anisometropia, with higher specificity. Matta & Silbert found a sensitivity of 98% and specificity of 88% for their criteria [15]. Arthur et al. found a lower sensitivity of 83% and higher specificity of 95% [16]. Singman et al. evaluated the performance of the Plusoptix applying multiple criteria, including the Matta & Silbert, ABCD, and Arthur criteria [21]. Matta & Silbert’s was highly sensitive (98%) but less specific (68%), while Arthur’s and ABCD’s criteria were both sensitive (86%, 92% respectively) and specific (85%, 90% respectively). Others also compared Matta & Silbert and Arthur’s criteria and found a high sensitivity (99%) and low specificity (47%) for the Matta & Silbert criteria, while the Arthur criteria had less sensitivity (89%) and higher specificity (76%) [22]. In our study, the Matta & Silbert criteria had the highest referral rate (41.9%), while the Arthur and the ABCD criteria resulted in more moderate referral rates (28.2%, 17.9% respectively). The performance of the different criteria based on studies in the literature and the referral rates we found in our study are explained by the different cut-off limits used for each refractive error. The Matta & Silbert set of criteria had the lowest thresholds for all refractive errors (except anisometropia) which subsequently led to high sensitivity and the highest referral rates for all refractive errors (except anisometropia) in our study. In contrast, the Arnold Medium and Specific criteria with their higher specificity and lower sensitivity had the highest thresholds and hence lowest rates of refractive errors except in the case of hyperopia in our study. The AAPOS criteria had the highest threshold for and subsequently the lowest rate of hyperopia in our study, considering that the AAPOS criteria refer to cycloplegic examination failure rate. It is well known that in children, accommodation changes vastly under different viewing circumstances. This is particularly important for hyperopia and hyperopic astigmatism for which normal children clear the image with accommodation, but which shows greater magnitude when examined under cycloplegia. In our study, the comparison of different criteria, including those intended for cycloplegic refraction, when applied to raw photoscreener results, shows the significant effect of the choice of criteria on the rates of referral and the different refractive errors detected by the device. Given these results and the documented accuracy of age-based instrument specific criteria proposed in the literature [13], we recommend both manufacturers and users to adopt the new, specific, age-based instrument referral criteria to maximize the value of instrument-based screening. We also encourage Plusoptix to adopt specific age-based instrument referral criteria (less than or greater 4 years of age) in future software versions. The psychometric characteristics of the criteria used are critical to the interpretation of screening results.

Studies in the United States reported referral rates of 8–16% with astigmatism being the most common refractive error affecting 28.4–69%, followed by hyperopia (12.8–58%), and then myopia (9.2–21%) [6,23,24]. In Europe and the United Kingdom, studies showed referral rates of 5.6% to 15%, with significant hyperopia in 7.9% and astigmatism in 4.3% [25]. In the Middle East, multiple studies reported that astigmatism was the most common refractive error affecting 12.1% to 60%, followed by hyperopia (from 1.5% to 22%), and then myopia (from 0.7% to 17%). A referral rate of 11% was reported by a study done in Turkey [26–28]. Our group previously reported the vision screening results of 1102 children aged 2–6 years who were screened for ARFs in the pediatric clinics of a university hospital setting; results showed referral rate of 5.8%; refractive ARFs detected by the Plusoptix were hyperopia in 1.7%, astigmatism in 1.4% and myopia in 0.3% [17]. Among the children referred for examination, the distribution of the refractive errors as confirmed by cycloplegic refraction were hyperopia in 51%, astigmatism in 41% and myopia in 8% [12]. The referral rate in our current study was 14% in private schools, which is similar to that in European studies. The refractive error distribution (12% astigmatism, 5% myopia and 2% hyperopia) were more consistent with rates reported in Europe than those in the United States: this could be explained by the heterogeneous population in Lebanon and the large proportion of Lebanese expats living or born in Europe. In Lebanon, only a single previous report investigated amblyopia risk factors in a cohort of 935 school children aged of 5 and 18 years; visual acuity was solely tested, and “ametropia” was noted in 15.7% of the screened population, 70% of whom were unaware of their visual defect [29].

In 2020, and during the COVID-19 lockdown, restricted outdoor activities and high exposure to digital screens accelerated myopia progression by 30% [30]. In Chinese children, 10% increase in myopia occurred between 2019 and 2020 [10]. The half-year incidence rate of myopia in China was 8.5% before the lockdown and increased to 13.62% after the quarantine [31]. Accelerated myopia progression was noted worldwide in children with pre-existing myopia. In our institution, a higher myopic SE was found during the pandemic-related lockdown in school-aged children compared to previous years [9]. This highlights the importance of automated vision screening for the early detection of refractive errors of increasing burden in different countries.

In our previous study in a tertiary care center in Beirut in 2021, utilizing the same photoscreener as the current study, the referral rate was 5.7% with hyperopia being the most common refractive error detected (1.7%), followed by astigmatism (1.4%) and then myopia (0.3%) [17]. In this community and school-based study done in Beirut, the referral rate was 22%. Referral rate was 36% and 14% in public and private school children, respectively. The rate of myopia was higher than hyperopia, which contrasts with the previous hospital-based screening results. The dominance of hyperopia in the previous study and astigmatism/myopia in the current study can be attributed to the difference in the sample size and age group studied. It is a fact that the prevalence of hyperopia is higher in younger children while myopia tends to become more prevalent in older age groups. The current study included 308 subjects while the previous study had 1102 children. The previous study included mostly healthy children 2–6 years old visiting a well-child clinic, while the current study included school children most of whom are 4 years or older. The current study was also done after the COVID-19 lockdown, which may have also influenced the differential rates of refractive error [9].

Our study detected significant differences in rates of ARF between public and private schools in the same city, with public schools being at a disadvantage. Studies in the literature showed that visual impairment and decreased access to pediatric eye care were correlated with decreased socio-economic status [32,33]. Studies across different countries revealed varying prevalence of refractive errors in children: Nigeria reported 2.1% (predominantly myopia, 1.9%), rural southern China noted much higher rates (myopia 36.8%, hyperopia 1%), while Nepal found refractive errors in 11.9% of children with no significant differences between public and private schools in Nepal [34–36]. Our study showed a higher referral rate of 36% in public schools vs 14% in private schools and, similarly, higher average SE. In addition, only 4.9% of those needing referral were wearing glasses in public schools as compared to 92.8% in private schools. The differences noted between school types in our study might be attributed to the lower socioeconomic status in public schools where vision screening is not financially accessible to families. In addition, systemic comorbidities are often undiagnosed in this population due to financial constraints. This emphasizes the importance of early vision screening especially in the public schools setting.

Our study has several strengths. It assessed the rate and type of refractive errors in school-aged children in Lebanon using an automated photoscreener, conducted in 2 private and 2 public schools to have a representative population of the community. We included more than 300 children and followed the AAPOS criteria for age-based referral, and while comparing them to five other validated criteria. However, we acknowledge some limitations including the limited sample size (coming from a small country like Lebanon) and the location of the four schools in one geographic area (Beirut) in Lebanon. The population included only young children, 3–6 years old, with the aim of targeting the age when amblyopia is still amenable to treatment. Additionally, children who needed referral did not undergo a comprehensive eye examination and cycloplegic refraction. Future larger scale studies are planned to evaluate the feasibility of integrating a comprehensive cycloplegic examination with school screenings to better report the prevalence rate of refractive errors and the actual number of children who ultimately need glasses, with sensitivity and specificity values. This approach would improve the validity of screening outcomes and provide a clearer understanding of referral accuracy and prevalence rates.

## Conclusions

Photoscreener referral rate and the detection of the different types of refractive errors varies significantly according to the referral criteria. Applying AAPOS age-based confirmatory exam failure criteria to the Plusoptix, we had a referral rate of 22%. The ABCD criteria had a similar rate of 17.9%. The Arthur exam failure criteria (28.2%) and Matta & Silbert criteria (41.9%) had higher rates, while the Arnold Medium and Specific criteria had the lowest referral rates (11.4% and 9.1%). The significant differences in referral rates found when applying different criteria underscores the importance of considering the psychometric characteristics of the chosen criteria when interpreting screening results. Based on our results and previous literature, we encourage users and manufacturers to adopt device specific age-based criteria. In our cohort, the most common refractive error for all criteria was astigmatism. According to the AAPOS criteria, the referral rate, all types of refractive errors, as well as the overall SE, were found to be higher in public schools in comparison to private schools. This highlights the importance of early vision screening, especially in the public sector.
